# The intolerance to functional genetic variation of protein domains predicts the localization of pathogenic mutations within genes

**DOI:** 10.1186/s13059-016-0869-4

**Published:** 2016-01-18

**Authors:** Ayal B. Gussow, Slavé Petrovski, Quanli Wang, Andrew S. Allen, David B. Goldstein

**Affiliations:** Institute for Genomic Medicine, Columbia University, New York, NY USA; Program in Computational Biology and Bioinformatics, Duke University, Durham, NC USA; Department of Medicine, The University of Melbourne, Austin Health and Royal Melbourne Hospital, Melbourne, VIC Australia; Department of Biostatistics and Bioinformatics, Duke University, Durham, NC USA

**Keywords:** RVIS, Intolerance, subRVIS, subGERP, Domains, Exons, Pathogenic

## Abstract

Ranking human genes based on their tolerance to functional genetic variation can greatly facilitate patient genome interpretation. It is well established, however, that different parts of proteins can have different functions, suggesting that it will ultimately be more informative to focus attention on functionally distinct portions of genes. Here we evaluate the intolerance of genic sub-regions using two biological sub-region classifications. We show that the intolerance scores of these sub-regions significantly correlate with reported pathogenic mutations. This observation extends the utility of intolerance scores to indicating where pathogenic mutations are mostly likely to fall within genes.

**Electronic supplementary material:**

The online version of this article (doi:10.1186/s13059-016-0869-4) contains supplementary material, which is available to authorized users.

## Background

We previously introduced the Residual Variation Intolerance Score (RVIS) [1], a framework that ranks protein-coding genes based on their intolerance to functional variation, by comparing the overall number of observed variants in a gene to the observed common functional variants. The basic idea behind this approach is the same as that behind approaches using phylogenetic conservation that rank genes by the degree to which they are evolutionarily conserved, except using standing human genetic variation to identify genes in which functional variation is strongly selected against and thus likely to be deleterious. This approach proved successful in prioritizing genes most likely to result in Mendelian disease [1]. Using the gene as the unit of analysis however fails to represent the reality that pathogenic mutations can often cluster in particular parts of genes.

**Table 1:** AIC comparisons of different sets of predictors

Predictor subset 1 (AIC)	Predictor subset 2 (AIC)	Minimal AIC	*P*
Base (20390.414)	subRVIS (20373.159)	subRVIS	0.0002
Base (20390.414)	subGERP (20370.726)	subGERP	5.3 × 10^–5^
subGERP (20370.726)	subRVIS (20373.159)	subGERP	0.296
subGERP (20370.726)	subRVIS + subGERP (20359.652)	subRVIS + subGERP	0.004
subRVIS (20373.159)	subRVIS + subGERP (20359.652)	subRVIS + subGERP	0.001

This table contains the AIC comparisons between different sets of predictors. All models contain the mutation rate as a covariate (Methods). Entries labeled ‘base’ indicate models using only the mutation rate and no other predictors. *P* is the probability that the model with the larger AIC minimizes the information loss from the model with the lower AIC

While there are many approaches that assess various characteristics of variants [2–4] which can in turn be used to try and determine whether or not a variant is likely to be pathogenic, current approaches to the problem of localizing pathogenic variants within sub-regions of a gene rely heavily on conservation to define important boundaries. The thought behind this is that more conserved regions within a gene are more likely to contain pathogenic variants. Another option to define genic sub regions is to utilize the functional information about the corresponding protein from databases of manually annotated proteins, such as Swiss-Prot [5]. In fact, some variant level predictors, such as MutationTaster [2], take these data into account when they are available. However, while ideally an approach that focused on parts of proteins would use divisions that correspond to functionally distinct parts of proteins, this information is not yet comprehensively available.

Here, we take a first step at an approach to divide the gene into sub-regions and rank the resulting sub-regions by their intolerance to functional variation. We use two divisions as surrogates for functionally distinct parts of the protein. The first is a division into protein domains, defined by sequence homology to known conserved domains. The second is a division into exons, reflecting that a gene can encode different isoforms of the protein using different exonic configurations.

For the protein domain division, we annotate each gene’s protein domains based on the Conserved Domain Database (CDD) [6], a collection of conserved domain sequences. The coding region of each gene was aligned to the CDD. The final domain coordinates for each gene were defined as the regions within the gene that aligned to the CDD and the unaligned regions between each CDD alignment.

Following this, we sought to create a ranking of the resulting sub-regions that would reflect their intolerance to functional variation. One common approach to this is to rank stretches of sequence by their phylogenetic conservation [7]. However, relying on conservation alone can fail to capture human specific constraint. Thus, we used the RVIS approach introduced in [1] to rank these regions solely based on human polymorphism data. We therefore generated the RVIS as described in [1], but now applied to the sequence stretches encoding the protein domains as the unit of analysis. As in [1], the scores were generated based on the NHLBI Exome Sequencing Project (ESP) exome variant calls [8]. This resulted in a genome-wide ranking of all domain encoding regions. To reflect its focus on sub-regions of genes, we term this overall approach sub-region Residual Variation Intolerance Scores (subRVIS). The subRVIS scores derived from this particular division into protein domains are designated domain subRVIS. We then repeated this for the exonic division, generating a set of exonic scores termed exon subRVIS. Following the original RVIS formulation, a lower subRVIS score indicates a more intolerant region.

As this approach is solely based on variation in the human population, we also constructed comparable conservation-based scores for both gene sub-divisions. We based our conservation approach on GERP++ [7], a method that assigns each genomic position a score denoting its estimated evolutionary constraint. In this approach, for each sub-region we calculate the average GERP++ score across its bases. We term this approach subGERP. We applied subGERP to the domain regions, and term the resulting scores domain subGERP. We repeated this for the exonic coordinates, and term the resulting scores exon subGERP. A higher subGERP score indicates an overall more conserved region.

To assess these scores, we developed a model for testing the utility of these scores in predicting the presence of previously reported pathogenic variants within these sub-regions. We show that domain subRVIS, domain subGERP, exon subRVIS, and exon subGERP are all significantly correlated with the presence or absence of pathogenic mutations within their corresponding regions. Further, we show that by dividing the gene into sub-regions we add useful information beyond the undivided genic RVIS score.

## Results and Discussion

### Region definitions and score generation

We defined each gene’s protein-coding region based on the consensus coding sequence project (CCDS) [9]. We divided these regions into domains based on the CDD [6] (Methods). The CDD is a collection of conserved domain sequences, represented as position-specific score matrices (PSSMs). Each gene’s coding sequence was aligned to CDD, using RPS-BLAST. In total, we annotated 8,988 different types of domains in 16,611 genes, covering 41.5 % of coding regions. The final domain coordinates for each gene were defined by both the regions of the coding sequence that aligned to the CDD and the unaligned regions between CDD alignments. These coordinates are available in Additional file 1. Using these coordinates, there are 89,522 regions in total, an average of five regions per gene. We calculated intolerance scores for these regions using the approach described in [1] and designated these scores domain subRVIS. As the division into exons is biologically relevant, in particular with respect to the splicing machinery, we also generated subRVIS scores whereby each exon constitutes a region, and termed these exon subRVIS (Additional file 2).

### Score assessment frameworks

Following the generation of the scores, we developed two frameworks to test how well regional intolerance scores can predict the distribution of known pathogenic variants in disease-associated genes (Methods). We use information for reported pathogenic variants from two large databases: ClinVar [10] (accessed June 2015) and the human gene mutation database (HGMD) [11] (release 2015.1). It is likely that in some cases only a portion of the gene was sequenced to detect pathogenic variants reported in these databases. This is unlikely to affect the results of our test because even in such scenarios, at the time of sequencing it was unknown which regions had more intolerant subRVIS scores, and thus the sequencing efforts would not be preferentially biased towards sequencing of subRVIS intolerant regions. We also limited the reported pathogenic variants to missense variants that were not adjacent to a canonical splice site (within one codon) and not predicted to cause loss of function (LoF) (Methods), as LoF variants, with some exceptions, generally damage the function of the entire protein indiscriminative of where within the protein they occur.

Our first framework is a gene-by-gene assessment of whether regional intolerance scores can predict the distribution of known pathogenic variants within each gene (Methods). Given that some genes have very few reported pathogenic variants or very few sub-regions, and that this test is applied separately to each gene, this test has limited power to detect significance. We limited our dataset to genes with at least two regions and at least one reported pathogenic variant (2,888 genes in domain subRVIS, 2,910 genes in exon subRVIS; Additional file 3 and Additional file 4).

Our second framework tests whether regional intolerance scores can predict the presence of known pathogenic variants on a genome-wide scale. This test is limited to the subset of genes for which we have reported pathogenic variants (3,046 genes; Additional file 5). Though this assessment is limited by our test data and does not cover all genes, this assessment can be used as an indicator to how well we expect regional intolerance scores to predict the overall distribution of pathogenic variants, and not just those that have been reported in our test data.

### Gene-specific testing

For the gene-specific testing, we focused our analyses on genes with at least two regions and at least one reported pathogenic variant. In domain subRVIS, we were able to assess 2,888 genes (Additional file 3). For 182 of the 2,888 genes (6.3 %), we found a significant relationship between domain subRVIS and the distribution of pathogenic variants (α = 0.05, false discovery rate (FDR); Additional file 3).

We ran the same assessment across the exon subRVIS scores. Here, we were able to assess 2,910 genes (Additional file 4). For 102 of the 2,910 genes (3.5 %), we found a significant relationship between exon subRVIS and the distribution of pathogenic variants (α = 0.05, FDR; Additional file 4).

**Fig. 1:**
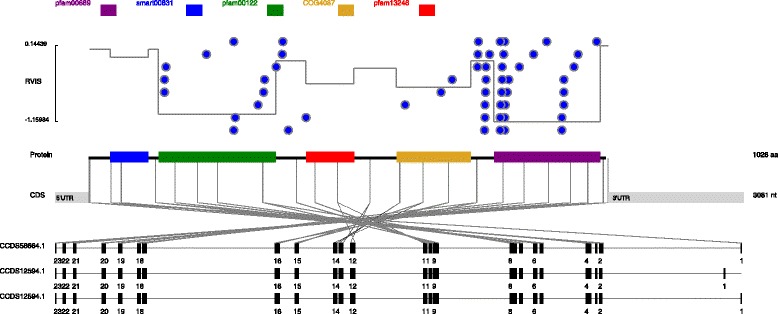
Distribution of reported pathogenic variants in *ATP1A3*. This figure shows the distribution of reported variants in *ATP1A3*. Each CDD conserved domain type is annotated in a different color. The Y axis represents the domain subRVIS scores. Each reported variant is marked with a blue circle

For these 182 genes where domain subRVIS predicts where mutations are found, there are many different patterns represented. In some cases, genes are somewhat evenly divided in more and less tolerant regions. One example in this category is the *ATP1A3* gene (Fig. 1). Overall, *ATP1A3* is a highly intolerant gene [1], which has been previously implicated with alternating hemiplegia of childhood and rapid-onset dystonia–parkinsonism [12, 13]. It falls in the 3rd percentile of the overall genic intolerance scores. *ATP1A3* has roughly two intolerance levels. The two most intolerant regions have intolerance scores of just below –1. These regions occupy 43 % of *ATP1A3*’s coding region. The remaining regions are more tolerant, with scores ranging from –0.573 to 0.144, with an average score of –0.145. Interestingly, these more tolerant regions carry far less previously identified pathogenic mutations (Fig. 1).

**Fig. 2:**
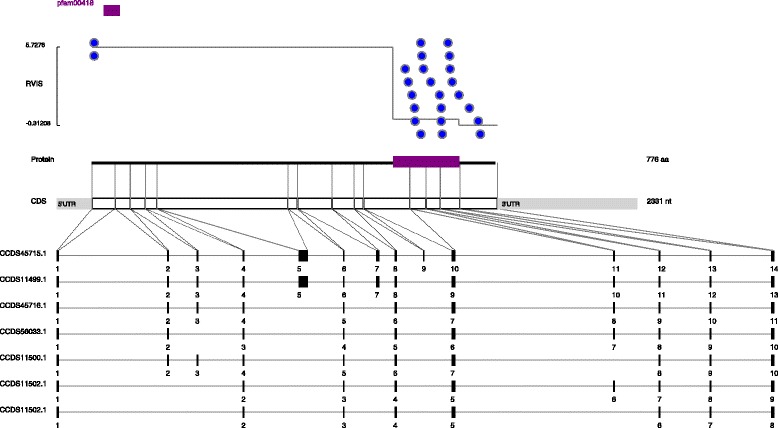
Distribution of reported pathogenic variants in *MAPT*. This figure shows the distribution of reported variants in *MAPT*. Each CDD conserved domain type is annotated in a different color. The Y axis represents the domain subRVIS scores. Each reported variant is marked with a blue circle

Some genes however show a far more extreme pattern. Overall, the *MAPT* gene (Fig. 2) is highly tolerant (98th percentile) despite carrying mutations that cause frontotemporal dementia [11, 14]. In fact, a small proportion (26 %) is very intolerant relative to the majority of the gene.

Strikingly, nearly all the reported pathogenic *MAPT* variants fall within two small intolerant sub-regions of *MAPT*. The third region is tolerant to variation, and therefore driving the overall genic intolerance score up despite the clear presence of a portion of the gene that causes disease when mutated. Though a fraction of the reported pathogenic variants is from publications that only sequenced exons falling in the two intolerant sub-regions [11], even when those variants are discounted the gene-specific test *P* value for *MAPT* remains unchanged and enrichment of reported pathogenic variants falling in the two intolerant regions remains clear (Fig. 2, FDR *P* value: 0.002).

### Genome-wide testing

Encouraged by the fact that subRVIS can, for at least some genes, clearly predict where within disease-associated genes pathogenic mutations are found, we sought to assess the genome-wide prediction of the regional intolerance scores. To this end, we implemented a logistic regression model to test how well regional intolerance scores can predict the presence of reported pathogenic variants within each sub-region genome-wide. For each set of intolerance scores tested, we also generated and tested 1,000 negative test sets. The comparison of the true *P* value to the negative set *P* values is termed the resampling *P* value (Methods). We restricted the test to the subset of genes that carry reported pathogenic variants (3,046 genes, Additional file 5). Chromosomes Y and MT were not assessed.

Overall, we found domain subRVIS to be predictive of the presence of pathogenic variants within domain encoding regions (*P* value: 1 × 10^–5^, resampling *P* value: 0.001, score effect size: –0.08, 3,046 genes).

We further wanted to verify that we were not recapturing the overall genic RVIS scores and confirm that dividing the gene into domain sub-regions is indeed adding information. We created a score vector in which each domain sub-region is assigned its gene’s overall genic RVIS score in place of its localized domain subRVIS score. We assessed this genic score vector across the subset of genes for which we have both subRVIS and RVIS scores (Additional file 5), and found that while genic RVIS is not predictive in this framework, subRVIS remains predictive for the subset of genes for which we have both subRVIS and genic RVIS scores (genic RVIS *P* value: 0.137, resampling *P* value: 0.142, score effect size: 0.03, 2,874 genes; domain subRVIS *P* value: 0.0002, resampling *P* value: 0.01, score effect size: –0. 07, 2,874 genes).

To assess the relationship between domain subRVIS and phylogenetic conservation, we similarly constructed a conservation score for each domain (domain subGERP). The Pearson’s correlation coefficient between domain subGERP and domain subRVIS is –0.204 (*P* value: <2.2 × 10^–16^; 95 % confidence interval: (–0.21, –0.197)). We found that domain subGERP is also predictive in our testing framework (*P* value: 3.5 × 10^–6^, resampling *P* value: 0.001, score effect size: 0.09, 3,046 genes). Furthermore, in a joint model with both domain subRVIS and domain subGERP, we found that both domain subRVIS and domain subGERP remain predictive (domain subRVIS *P* value: 0.0003, score effect size: –0.07; domain subGERP *P* value: 8.7 × 10^–5^, score effect size: 0.07; 3,046 genes), indicating that both scores add significant independent information about the localization of pathogenic variation.

To formally test the contribution of these different scores, we calculated and compared the Akaike information criterion (AIC) of the above model, using different predictor combinations of the two significant scores (subRVIS and subGERP). Based on these comparisons neither subRVIS nor subGERP appear to be a significantly stronger predictor (Table 1). We further found that including both subRVIS and subGERP minimizes information loss beyond subGERP alone (*P* value: 0.004) and beyond subRVIS alone (*P* value: 0.001).

The results above show that analyzing the intolerance of regions of genes corresponding to protein domains can provide significant information of where disease causing mutations are likely to be found. However, this does not itself indicate that the use of the protein domains adds information. In fact, it could be the case that any sub-divisions of genes of similar size to protein domains would allow such prediction. More generally, of course, it could be that other ways of sub-dividing genes could be even more informative.

To explore these questions, we first tried to determine whether dividing the genes into biological domains adds information beyond dividing genes into similarly sized sub-regions without biological information. We permuted the CDD domains within each gene randomly, and generated domain subRVIS scores for each of these random permutations (Methods). We then assessed the prediction of these scores using the same model we used for the original scores. We repeated this 100 times, and created a distribution of the effect sizes of subRVIS scores across the random permutations. While we expect that many, or possibly all, of these divisions will be significantly predictive, we sought to test whether we have significantly more information in the biological division. The domain division score effect size has a larger absolute value than 99 out of the 100 permuted division score effect sizes. Thus, incorporating biological information does seem to contribute useful information beyond simply considering randomly assigned parts of the gene, when the units correspond in size to protein domains (permutation *P* value: 0.02; Additional File 6: Figure S1).

Next, we sought to explore whether the exonic division might do better than the division into regions of genes corresponding to protein domains. With the exonic division, Pearson’s correlation coefficient between exonic subRVIS and exonic subGERP is -0.126 (p-value: <2.2 × 10^-16^; 95 % confidence interval: [-0.130, -0.121]). We found that using intolerance scores at the level of the exon is also predictive of the presence of pathogenic variants within exons (p-value: 0.0001, resampling p-value: 0.001, score effect size: -0.04, 3046 genes). Further, we found that exon subGERP is also predictive in this framework (p-value: 7.8 × 10^-16^, resampling p-value: 0.001, score effect size: 0.09, 3046 genes).

Similar to our analysis of domain encoding regions, this does not itself indicate that the use of the biological parts is actually adding information – it could be that simply considering parts of genes would allow comparable performance. Thus, just as we did with domains, we generated 100 sets of coordinates in which we permuted the exons within each gene randomly and generated exonic subRVIS scores for these shuffled exons (Methods). We then assessed the prediction of these scores. The exon division score effect size has a smaller absolute value than all but one of the permuted division score effect sizes. Thus, it appears that incorporating the exonic information may not contribute useful information beyond other divisions in which the units correspond in size to exons (Additional File 6: Figure S2).

### Examining the relationship with variant level scores

Although the subRVIS approach does not constitute a variant level predictor, we wanted to verify that the information we were gaining from subRVIS was independent from the information already available from commonly used variant level predictors. Thus, we sought to explore the relationships between domain subRVIS and three variant level predictors: MutationTaster [2], PolyPhen-2 [4], and CADD [3]. We based our assessment on a set of 250,000 simulated variants (Additional file 7) within the domain subRVIS coordinates. The full results of this assessment can be found in Additional file 8.

We found that neither PolyPhen-2 nor CADD strongly correlated with domain subRVIS, with Pearson’s correlation coefficients of –0.0548 and –0.0811, respectively. The negative correlation is expected, as lower subRVIS scores indicate more intolerant regions and higher PolyPhen-2 or CADD scores indicate more damaging variants.

We converted MutationTaster’s predictions into scores on a scale of 0 to 1, with 0 corresponding to predicted pathogenic and 1 corresponding to predicted non-pathogenic (Methods). The Pearson’s correlation coefficient with the MutationTaster scores is 0.159. Thus, domain subRVIS and MutationTaster are correlated to a higher degree than domain subRVIS and either of the other two scores (PolyPhen-2 and CADD). This is not unexpected, as MutationTaster does consider protein domain information when it is available.

Given this overlap, we sought to explore the relationship between the MutationTaster predictions and domain sub-regions where pathogenic variants have been previously reported. We divided the MutationTaster variant scores based on whether the corresponding simulated variant falls in a domain sub-region that has previously reported at least one pathogenic variant (n = 20,382 simulated variants) or not (n = 228,116 simulated variants). We compared these two distributions of scores and found that they differ (*P* value: 2.48 × 10^–256^, Wilcoxon rank sum test), with lower (more likely pathogenic) variant level MutationTaster scores corresponding to variants identified in disease-associated regions.

### Application to patient data

We have previously shown that neuropsychiatric case populations show an enrichment of *de novo* mutations that have a damaging (≥0.95) PolyPhen-2 score [4] and occur in an intolerant (≤25th percentile) genic RVIS gene when compared to *de novo* mutations found among controls [1]. The idea behind this multi-tiered approach that includes both variant level and regional level prioritizations is that the interpretation of a variant’s effect is more informative when it is known if the variant falls in a region that is depleted of functional variation. Thus, if a variant is likely damaging to the protein and also affects an intolerant gene, it is more likely to be pathogenic and therefore we expect an enrichment of these in cases. We designated mutations fitting these criteria as ‘hot zone’ mutations.

We applied this approach using the subRVIS scores in place of the genic RVIS scores on two case datasets: *de novo* mutations in autism [15] and in epileptic encephalopathies [16]. For controls, we used the controls provided in [15] as controls for both sets of cases (Methods).

We found that within the epilepsy cohort, 77 out of 366 (21 %) of the *de novo* mutations were subRVIS hot zone mutations, while only 212 out of 1345 (16 %) of the *de novo* mutations in controls were (Fisher’s exact test *P* value: 0.018). Using the same test with the genic RVIS scores gave a stronger signal (Fisher’s exact test *P* value: 0.001). In the autism cohort, we found a strong signal for genic RVIS (Fisher’s exact test *P* value: 0.0001) and an insignificant subRVIS score signal (Fisher’s exact test *P* value: 0.275).

Despite the fact that, for now, genic RVIS is still more predictive, we are encouraged by the fact that we can still detect hot zone *de novo* mutation enrichment with a focus on sub-regions. This is especially impressive given the smaller size of the subRVIS regions. As the number of available control reference cohorts grows, the resolution of the subRVIS score is anticipated to improve.

## Conclusions

Despite its introduction only 2 years ago, it is already clear that consideration of genic intolerance provides a valuable new dimension in the interpretation of patient genomes. Intolerance scores have been used repeatedly to interpret observations of mutations in patients with unresolved or undiagnosed diseases [17–22] and have been used to interpret *de novo* mutations across a broad range of diseases [23–26].

Despite this promise, the gene as the unit of analysis is coarse. Here we have shown that sub-dividing genes into regions corresponding to protein domains or regions of the size of exons can provide significant information about where in disease causing genes pathogenic mutations are most likely to be found. There are a number of ways we expect this added resolution to be useful in interpreting genomes. An obvious example is to focus attention on mutations that occur in genes that are not intolerant overall, but that occur in a particularly intolerant region of the gene. The reverse pattern is also important. One of the most challenging aspects of the interpretation of personal genomes today is the high percentage of false positive mutations in disease databases, and the fact that these mutations clearly have higher population allele frequencies than the true positives. It is very likely that these false positive mutations are preferentially drawn from the more tolerant regions of genes that cause disease, giving us a possible new pointer to candidate false positive mutations.

To be alert to such possibilities, we have created an online tool for plotting variants across domain sub-regions within a gene (www.subrvis.org). This tool can help researchers explore which domains within a gene their variants of interest fall in and what the corresponding subRVIS scores are. We further constructed a score to reflect the degree to which genes vary in intolerance among their regions. The expectation is that in some genes the intolerance to variation will be uniform across its sub-regions, while in others the intolerance to variation will vary greatly across its sub-regions. Thus, this score was constructed by calculating, per gene, the standard deviation of its domain subRVIS scores. Only genes with at least three domain sub-regions were assessed. Though these scores can be useful in predicting whether we expect pathogenic mutations to cluster in specific sub-regions within a given gene, currently there is not a relationship between these scores and whether known pathogenic mutations actually do so. These scores are available in Additional file 9.

One important point to emphasize is that the minimum unit size that can be effective in an RVIS framework depends critically on the number of individuals that have been sequenced in the reference cohort, since the ability to distinguish different genomic regions depends on observing variation in those regions. While our research shows the utility of the basic subRVIS approach, the power of this approach will steadily increase as the number of sequenced individuals increases.

The research presented here demonstrates the importance of accounting for protein domains in human disease studies. In particular, quantifying a gene’s domains’ intolerance to variation has utility in identifying causal variants. We anticipate that our methodology will continue to improve as we gain access to more sequencing data. There are other ways, outside of conserved domains, to divide a protein into domains, such as tertiary structure. Future approaches can incorporate these and other annotations to divide proteins into biologically relevant sub-regions.

## Methods

The software used in this publication is available on GitHub (https://github.com/igm-team/subrvis), released under the MIT license.

### Defining the domains

We define each gene’s protein-coding sequence based on its Consensus Coding Sequence (CCDS release 15, accessed November 2013) entry [5]. In order to avoid multiple gene definitions, genes with multiple CCDS transcripts are assigned the gene’s canonical transcript, as this is the longest and most encompassing transcript. Next, each gene’s CCDS entry is translated into protein sequence and aligned to CDD (version 3.11) [6] using RPS-BLAST (version 2.2.28+). No multi-domains in CDD were used, to avoid grouping multiple single domains into one sub-region in our domain definitions. Only alignments with maximal E-value of 1e-2 were considered. When two domains overlapped in the RPS-BLAST results, the domain with the better alignment score was kept.

### Calculating subRVIS Scores

subRVIS scores were calculated for the domains, exons, 100 permuted domains, and 100 permuted exons. The scores were calculated using the NHLBI Exome Sequencing Project (ESP) [8], as described in [1]. Each position in each sub-region is first checked for adequate coverage. Only positions with ≥10× average coverage in the ESP were considered. ESP variant calls were further filtered to only retain variants with a ‘PASS’ filter status. Following this, using the ESP variant calls that qualify based on both these criteria, the tally of all variants per each sub-region is regressed against the count of common (>0.1 % minor allele frequency) non-synonymous variants in the sub-region, as per [1]. The studentized residual of each sub-region is its score. Thus, the subRVIS score quantifies the departure of the observed number of common non-synonymous variants from the expectation given the total number of variants in each genomic region. One of the outcomes of using the residuals from this regression as the score is that they are, by definition, orthogonal to the tally of all variants in their corresponding sub-region and thus orthogonal to the overall distribution of variants in that genomic region. A total of 89,335 domain subRVIS scores (Additional file 10) and 185,355 exon subRVIS scores (Additional file 11) were generated. Y and MT chromosome genes were not assessed.

### Score prediction test dataset

To test the utility of subRVIS scores in predicting which regions are more likely to carry pathogenic variants, we required a database of known pathogenic variants. We combined data from ClinVar (accessed June 2015) [10] and HGMD (release 2015.1) [11], filtering for ClinVar entries labeled ‘Pathogenic’ and HGMD entries tagged as ‘DM’ (disease causing mutation).

We then ran Variant Effect Predictor (version 73) [27] and filtered for canonical variants that were labeled as ‘missense_variant’ and were not labeled as any of the following: ‘incomplete_terminal_codon_variant’, ‘splice_region_variant’, ‘stop_gained’, and ‘stop_lost’.

### Calculating mutation rates

As we are testing against raw counts of pathogenic mutations, we required a covariate to account for the difference in counts that are due to sequence mutability and unrelated to intolerance. For this, we calculated the mutation rates for each sub-region (Additional file 10 and Additional file 11) based on its sequence composition [23]. This calculation is not based on any other data used in this manuscript.

### Regional score gene-specific prediction test model

To test whether the subRVIS scores are predictive at the single gene level, we designed and implemented a permutation test that predicts the distribution of reported pathogenic variants within a single gene using a set of scores. Genes with less than two regions or less than one reported pathogenic variant were not assessed.

Let *n*_*g*_ be the number of regions in gene $\mathit{\mathsf{g}}$. Let ${\mathit{\mathsf{Y}}}_{\mathit{\mathsf{g}}}$ be a vector of length *n*_*g*_ containing the counts of reported pathogenic variants across sub-regions in gene $\mathit{\mathsf{g}}$. Let ${\mathit{\mathsf{Z}}}_{\mathit{\mathsf{g}}}$ be a vector of length *n*_*g*_ containing the mutation rates across sub-regions in gene $\mathit{\mathsf{g}}$, based on sequence composition [23]. Let ${\mathit{\mathsf{X}}}_{\mathit{\mathsf{g}}}$ be a vector of length *n*_*g*_ containing the intolerance scores across sub-regions in gene $\mathit{\mathsf{g}}$.

For each gene, we then calculated the expected distribution of pathogenic variants within the gene based on the sub-region mutation rates and the total number of reported pathogenic variants within the gene:

$$ {E}_{\mathsf{g}i}=\left(\sum_i^{n_g}{Y}_{\mathsf{g}i}\right)\ast \left(\frac{Z_{\mathsf{g}i}}{{\displaystyle {\sum}_i^{n_g}}{Z}_{\mathsf{g}i}}\right). $$

Thus, ${\mathit{\mathsf{E}}}_{\mathit{\mathsf{g}}}$ is a vector of the expected number of reported pathogenic variants in each sub-region based on the gene’s mutation rates and total number of reported pathogenic variants.

For each sub-region we subtracted the expected number of pathogenic variants $\left({\mathit{\mathsf{E}}}_{\mathit{\mathsf{g}}}\right)$ from the observed number of pathogenic variants $\left({\mathit{\mathsf{Y}}}_{\mathit{\mathsf{g}}}\right)$. This vector denotes the departure of each sub-region’s tally of reported pathogenic variants from its expected tally. This vector is designated ${\mathit{\mathsf{D}}}_{\mathit{\mathsf{g}}}$.

We calculated a score per gene, designated ${\mathit{\mathsf{C}}}_{\mathit{\mathsf{g}}}$, denoting both the departure of the reported pathogenic variants distribution from the expectation and the relationship with the intolerance score:

$$ {C}_{\mathsf{g}}=cov\left({D}_{\mathsf{g}},{X}_{\mathsf{g}}\right). $$

We expect that a lower intolerance score will correspond with higher pathogenic variant counts than expected, and therefore we expect the covariance to be negative. To test this, we performed a permutation test, where each permutation has a different distribution of the reported pathogenic variants.

For each permutation, we drew the distribution of the reported pathogenic variants from a multinomial distribution, where the number of trials is the total tally of reported pathogenic variants for the gene and the probability for each sub-region is the fraction of the gene’s mutation rate that it occupies $\left(\frac{Z_{\mathsf{g}i}}{{\displaystyle {\sum}_i^{n_g}}{Z}_{\mathsf{g}i}}\right)$. Following this, we calculated the departure from expectation and the covariance as described above.

We repeated this *n*_*p*_  = 20,000 times. To test how the intolerance score prediction for the true distribution of pathogenic variants compares to the prediction for the permuted distributions, we counted how many times out of the *n*_*p*_  = 20,000 permutations the permuted covariance is smaller than or equal to the true covariance. This count is designated *G*. The permutation *P* value is calculated by the following equation: (*G* + 1)/(*n*_*p*_  + 1).

### Regional score genome-wide prediction test model

To test whether the regional scores are predictive at the level of the genome, we designed and implemented a model that predicts the presence or absence of reported pathogenic variants at each sub-region using a set of scores.

As the presence or absence of reported pathogenic variants in a sub-region will depend greatly on that sub-region’s mutability, what we are trying to determine in this model is whether our scores can predict the presence of pathogenic variation in a sub-region after accounting for the region’s mutability. We divide any score predictors (subRVIS, subGERP, genic RVIS) within this model by their standard deviation in order to allow for the interpretation of the effect sizes in terms of standard deviations.

Previously, we were considering a single gene indexed by *g*. Here, we are considering genome-wide analysis, therefore we dropped the *g *from notation. Specifically, let *n* be the number of sub-regions across all the genes in the genome. Let *Y* be a vector of length *n* with components (*Y*_*i*_ , *i* = 1,…, *n*) corresponding to each sub-region taking on either a 1 or a 0, respectively, denoting presence or absence of at least one non-LoF pathogenic variant within the sub-region. Let *Z* be a vector of length *n* containing the sub-regions’ mutation rates, based on sequence composition [23]. Let *X* be a vector of length *n* containing the sub-regions’ intolerance scores, scaled across all sub-regions by dividing each intolerance score by the standard deviation of all the sub-regions’ intolerance scores.

To evaluate the relationship between the score and the presence of pathogenic variants we fit the following logistic regression model:

$$ logit\left( \Pr \left({Y}_i=1\right)\right)=\alpha +{\beta}_1\ast \log \left({Z}_i\right)+{\beta}_2{X}_i, $$

where i=1,...,n.

Note that *β*_2_ captures the strength of the relationship between *X*, the intolerance scores, and *Y*, while adjusting for regional mutability. We refer to *β*_2_ as the ‘score effect size’ and report it in the text as a metric for how well a given model performs.

Next, we wanted to assess how the model performs on negative test sets. For each intolerance score genome-wide test we generated 1,000 resampled response vectors. To create the resampled response vectors, we resampled the 0 s and 1 s within the true response vector (*Y*) so that sub-regions with a larger mutation rate (*Z*) were more likely to be assigned a 1. Specifically, we resampled *Y* without replacement with sampling probabilities given by $\frac{Z}{{\displaystyle {\sum}_i^n}{Z}_i}$. The idea is that under neutrality the more mutable a region is the more likely it is to carry mutations. By using this resampling method, we preserve the number of regions containing pathogenic variants and therefore the number of zeros and ones in our response vector remains the same.

Using the same genome-wide assessment that we used for the observed data, we tested the intolerance scores’ prediction against each of the 1,000 resampled response vectors. This resulted in a vector of negative test set *P* values. To test how the observed set of reported pathogenic variants compares to that obtained by resampling, we enumerated, across all (*R* = 1,000) resampled datasets the number of times (*C*) the *P* value from the resampled data analysis is larger than the *P* value obtained from the observed data analysis. We define our resampling *P* value as: (*R* – *C* + 1)/(*R* + 1).

### AIC comparisons

To compare AICs between two models, we first identify the model with the lower AIC, representing the model estimated to have less information loss. We designated this AIC_min_ and we designated the other AIC as AIC_max_. To calculate the relative probability that AIC_max_ is the model that minimizes information loss (designated here as *p*), we calculate:

$$ p= \exp \left(\frac{AI{C}_{min}- AI{C}_{max}}{2}\right). $$

The resulting value indicates that the probability that AIC_max_ minimizes the information loss from AIC_min_ is *p*. A high *p* indicates that AIC_max_ may have less information loss than AIC_min_, while a low *p* indicates that it is unlikely that AIC_max_ minimizes information loss in comparison to AIC_min_.

### Region permutation test

To test our model on randomly permuted regions, we performed the following:

1. For each gene we took into account the sizes of each of its sub-regions.
2. We permuted the sizes of each gene’s sub-regions, resulting in a set of the same sub-regions in a random order.
3. We then re-divided each gene based on the permuted set of sub-regions. Thus, after the permutation each gene maintains the same number and size distribution of sub-regions as in the biological division.
4. For this permuted set of sub-regions, we generated sub-region intolerance scores, calculated the sub-region mutation rates and counted the number of pathogenic variants in each sub-region.
5. Following this, we tested prediction across the permuted set of sub-regions using the same genome-wide assessment that we used for the biological division.
6. We recorded the effect size of the intolerance scores in this assessment.
7. We repeated steps (1) through (6) 100 times.
8. This resulted in a vector of effect sizes that constitutes our null distribution of effect sizes for the permutation test.

To test how the biological division compares to the permuted divisions, we counted how many times out of the *n*_*p*_  = 100 permutations the absolute value of the permuted division score effect size is smaller than the absolute value of the biological division score effect size. This count is designated *X*. The permutation *P* value is calculated by the following equation: (*n*_*p*_  − *X* + 1) /(*n*_*p*_  + 1).

### GERP scores

To quantify phylogenetic conservation across sub-regions, we generated a novel vector for each sub-region division that simply reflects the average GERP++ [7] score (where available) for those coordinates (Additional file 10 and Additional file 11).

### MutationTaster scores

We ran MutationTaster’s QueryEngine (http://www.mutationtaster.org/StartQueryEngine.html, accessed September 2015) [2] with default options, outside of the option to filter against the 1000 Genomes project. By default, this option is selected. As we wanted analysis results for all variants, we deselected this option.

MutationTaster uses a Bayes classifier to determine whether a variant is a polymorphism or disease causing. The classifier has four output options:

1. disease_causing: probably deleterious.
2. disease_causing_automatic: known to be deleterious based on existing databases.
3. polymorphism: probably harmless.
4. polymorphism_automatic: known to be harmless based on existing databases.

Along with the prediction, for each variant MutationTaster outputs an estimated probability for the prediction. More information on MutationTaster can be found at http://www.mutationtaster.org/info/documentation.html or at [2].

To convert these results into a score between 0 and 1, we devised the following criteria:

1. If the prediction is polymorphism, use the probability as the score. This will always be above 0.5. Thus, predicted polymorphisms receive scores in the range of 0.5 to 1.
2. If the prediction is disease_causing, the score is the probability subtracted from 1. As the probability will always be above 0.5, the predicted disease causing variants receive scores in the range of 0 to 0.5.
3. If the prediction is either polymorphism_automatic or disease_causing_automatic, this indicates that the variant’s prediction is based on a database entry, not the Bayes classifier. If the Bayes classifier disagrees with the automatic prediction, the probability will be less than 0.5. In these instances, we reassigned the variant’s prediction to match the Bayes classifier’s and reassigned the probability to 1 minus the originally reported probability. Following this, we treated the variant as described above.

### Applying the hot zone approach

For both the autism [15] and epileptic encephalopathies [16] *de novo* mutations data we limited to single-nucleotide variants, falling in regions for which we have both subRVIS and genic RVIS scores. We calculated each variant’s PolyPhen-2 score using PolyPhen-2 HumVar [4]. Synonymous variants were assigned 0 while canonical splice, stop gain, and stop loss variants were assigned 1. Mutations present as variants in the NHLBI ESP exome variant calls [8] were excluded.

All the mutations in the epileptic encephalopathies data from the Epi4K study [16] are Sanger validated.

For the autism data from [15], we required that the mutations not be called in both siblings. We additionally required that either: (1) at least one of the institutes analyzing the data (Cold Spring Harbor Laboratory, Yale School of Medicine, University of Washington) had validated the mutation; or (2) at least one of the institutes labeled the mutation as a ‘strong’ variant call while no other institute labeled the mutation as ‘not called’ or ‘weak’.

### Estimate of the disease risk per protein domain

Given the potential interest in whether some CDD protein domain types are more likely to carry reported pathogenic mutations than others, we have created a table including the tally of reported pathogenic mutations and the cumulative mutation rate for each CDD protein domain type across genes (Additional file 12). This table also includes the tally divided by the cumulative mutation rate. This is meant to serve as an approximate estimate denoting the number of reported pathogenic mutations after controlling for mutation rate. For comparative purposes, a higher value indicates more reported mutations given the sequence context.

### Ethical approval

No ethical approval was required.

### Availability of data and materials

The data and materials used in this manuscript are either previously published or are available in this publication as Additional files 1, 2, 3, 4, 5, 6, 7, 8, 9, 10, 11, and 12. The software used in this publication is available on GitHub (https://github.com/igm-team/subrvis), released under the MIT license.

## Additional files

Additional file 1:
**A text file in BED format containing domain subRVIS sub-region boundaries.** Each sub-region name contains three fields separated by a colon - the gene name; the domain type, which is either the CDD PSSM-Id of the aligned domain or a ‘-’ denoting that no CDD domain was aligned to this region; the domain type again followed by an underscore, followed by the occurrence number of this domain type. (TXT 10178 kb) Additional file 2:
**A text file in BED format containing exon subRVIS sub-region boundaries.** Each sub-region name contains three fields separated by a colon. Although for the exon sub regions only two fields are required, we used three fields to maintain consistency with the domain sub region boundaries file. Therefore the second and third fields are equal to each other. The format is: the gene name; the letter ‘E’ followed by the exon number; the letter ‘E’ followed by the exon number again. (TXT 6710 kb) Additional file 3:
**A text file of the FDR adjusted** ***P*** **values per-gene for the domain subRVIS gene-specific tests across the subset of genes for which we have at least two regions and at least one reported pathogenic variant.** (TXT 35 kb) Additional file 4:
**A text file of the FDR adjusted** ***P*** **values per-gene for the exon subRVIS gene-specific tests across the subset of genes for which we have at least two regions and at least one reported pathogenic variant.** (TXT 35 kb) Additional file 5:
**A text file containing the subset of genes for which we have reported pathogenic variants (3,046 genes).** The columns denote whether or not we have subRVIS, RVIS, or subGERP scores for the gene in question. (TXT 53 kb) Additional file 6:
**A PDF containing Figure S1 and Figure S2, which show the distribution of score effect sizes from random permutations.** (PDF 195 kb) Additional file 7:
**A text file containing the set of 250,000 random variants generated for the variant level predictors comparisons with the corresponding variant prediction scores and disease-associated status (1 indicates that the domain region the variant falls in has at least one previously reported pathogenic variant, 0 indicates that it has no previously reported pathogenic variants).** (TXT 12240 kb) Additional file 8:
**A PDF containing the full results of the comparison to variant level predictors.** (PDF 88 kb) Additional file 9:
**A text file with the standard deviation of the domain subRVIS scores, per gene, across genes with at least three regions.** (TXT 671 kb) Additional file 10:
**A text file of domain: subRVIS scores, subGERP scores, genic RVIS scores, pathogenic counts, mutation rates, and coverage percentages.** (TXT 7982 kb) Additional file 11:
**A text file of exonic: subRVIS scores, subGERP scores, genic RVIS scores, pathogenic counts, mutation rates, and coverage percentages.** (TXT 15950 kb) Additional file 12:
**A text file with an estimate of the disease risk per protein domain.** The format of this table is: PSSM ID; domain name; tally of reported pathogenic mutations in this domain type across genes; cumulative mutation rate in this domain type across genes; the tally divided by the cumulative mutation rate. (TXT 405 kb)

## Abbreviations

CDD: Conserved domain database
FDR: False discovery rate
HGMD: Human gene mutation database
LoF: Loss-of-function
PSSM: Position-specific score matrix
RVIS: Residual Variation Intolerance Score
subRVIS: Sub-region Residual Variation Intolerance Score

## References

[1] Petrovski S, Wang Q, Heinzen EL, Allen AS, Goldstein DB (2013). Genic intolerance to functional variation and the interpretation of personal genomes. PLoS Genet.

[2] Schwarz JM, Cooper DN, Schuelke M, Seelow D (2014). MutationTaster2: mutation prediction for the deep-sequencing age. Nat Methods.

[3] Kircher M, Witten DM, Jain P, O’Roak BJ, Cooper GM, Shendure J (2014). A general framework for estimating the relative pathogenicity of human genetic variants. Nat Genet.

[4] Adzhubei IA, Schmidt S, Peshkin L, Ramensky VE, Gerasimova A, Bork P (2010). A method and server for predicting damaging missense mutations. Nat Methods.

[5] UniProt C (2015). UniProt: a hub for protein information. Nucleic Acids Res.

[6] Marchler-Bauer A, Zheng C, Chitsaz F, Derbyshire MK, Geer LY, Geer RC (2013). CDD: conserved domains and protein three-dimensional structure. Nucleic Acids Res.

[7] Davydov EV, Goode DL, Sirota M, Cooper GM, Sidow A, Batzoglou S (2010). Identifying a high fraction of the human genome to be under selective constraint using GERP++. PLoS Comput Biol.

[8] Exome Variant Server, NHLBI GO Exome Sequencing Project (ESP). Seattle, WA. http://evs.gs.washington.edu/EVS/. Accessed 3rd August 2012.

[9] Pruitt KD, Harrow J, Harte RA, Wallin C, Diekhans M, Maglott DR (2009). The consensus coding sequence (CCDS) project: Identifying a common protein-coding gene set for the human and mouse genomes. Genome Res.

[10] Landrum MJ, Lee JM, Riley GR, Jang W, Rubinstein WS, Church DM (2014). ClinVar: public archive of relationships among sequence variation and human phenotype. Nucleic Acids Res.

[11] Stenson PD, Mort M, Ball EV, Shaw K, Phillips A, Cooper DN (2014). The Human Gene Mutation Database: building a comprehensive mutation repository for clinical and molecular genetics, diagnostic testing and personalized genomic medicine. Hum Genet.

[12] Heinzen EL, Swoboda KJ, Hitomi Y, Gurrieri F, Nicole S, de Vries B (2012). De novo mutations in ATP1A3 cause alternating hemiplegia of childhood. Nat Genet.

[13] de Carvalho AP, Sweadner KJ, Penniston JT, Zaremba J, Liu L, Caton M (2004). Mutations in the Na+/K+ -ATPase alpha3 gene ATP1A3 are associated with rapid-onset dystonia parkinsonism. Neuron.

[14] Goedert M, Crowther RA, Spillantini MG (1998). Tau mutations cause frontotemporal dementias. Neuron.

[15] Iossifov I, O’Roak BJ, Sanders SJ, Ronemus M, Krumm N, Levy D (2014). The contribution of de novo coding mutations to autism spectrum disorder. Nature.

[16] EuroEPINOMICS-RES Consortium (2014). Epilepsy Phenome/Genome Project, Epi4K Consortium. De novo mutations in synaptic transmission genes including DNM1 cause epileptic encephalopathies. Am J Hum Genet..

[17] Chen YZ, Friedman JR, Chen DH, Chan GC, Bloss CS, Hisama FM (2014). Gain-of-function ADCY5 mutations in familial dyskinesia with facial myokymia. Ann Neurol.

[18] Enns GM, Shashi V, Bainbridge M, Gambello MJ, Zahir FR, Bast T (2014). Mutations in NGLY1 cause an inherited disorder of the endoplasmic reticulum-associated degradation pathway. Genetics Med.

[19] Hildebrand MS, Damiano JA, Mullen SA, Bellows ST, Oliver KL, Dahl HH (2014). Glucose metabolism transporters and epilepsy: only GLUT1 has an established role. Epilepsia.

[20] Homan CC, Kumar R, Nguyen LS, Haan E, Raymond FL, Abidi F (2014). Mutations in USP9X are associated with X-linked intellectual disability and disrupt neuronal cell migration and growth. Am J Hum Genet.

[21] Puskarjov M, Seja P, Heron SE, Williams TC, Ahmad F, Iona X (2014). A variant of KCC2 from patients with febrile seizures impairs neuronal Cl- extrusion and dendritic spine formation. EMBO Rep.

[22] Takata A, Xu B, Ionita-Laza I, Roos JL, Gogos JA, Karayiorgou M (2014). Loss-of-function variants in schizophrenia risk and SETD1A as a candidate susceptibility gene. Neuron.

[23] Epi4K Consortium, Epilepsy Phenome/Genome Project (2013). De novo mutations in epileptic encephalopathies. Nature.

[24] Gilissen C, Hehir-Kwa JY, Thung DT, van de Vorst M, van Bon BW, Willemsen MH (2014). Genome sequencing identifies major causes of severe intellectual disability. Nature.

[25] McCarthy SE, Gillis J, Kramer M, Lihm J, Yoon S, Berstein Y (2014). De novo mutations in schizophrenia implicate chromatin remodeling and support a genetic overlap with autism and intellectual disability. Mol Psychiatry.

[26] Zhu X, Need AC, Petrovski S, Goldstein DB (2014). One gene, many neuropsychiatric disorders: lessons from Mendelian diseases. Nat Neurosci.

[27] McLaren W, Pritchard B, Rios D, Chen Y, Flicek P, Cunningham F (2010). Deriving the consequences of genomic variants with the Ensembl API and SNP Effect Predictor. Bioinformatics.

