# Supplementary material for: The intolerance to functional genetic variation of protein domains predicts the localization of pathogenic mutations within genes
Source: Genome Biol. 2016 Jan 18;17:9. doi: 10.1186/s13059-016-0869-4 (PMC4717634; doi:10.1186/s13059-016-0869-4)

Figure S1. Distribution of score effect sizes from random domain permutations.

A histogram of the score effect size estimates from 100 random domain permutations. The true domain division score effect size estimate is marked as a red circle.

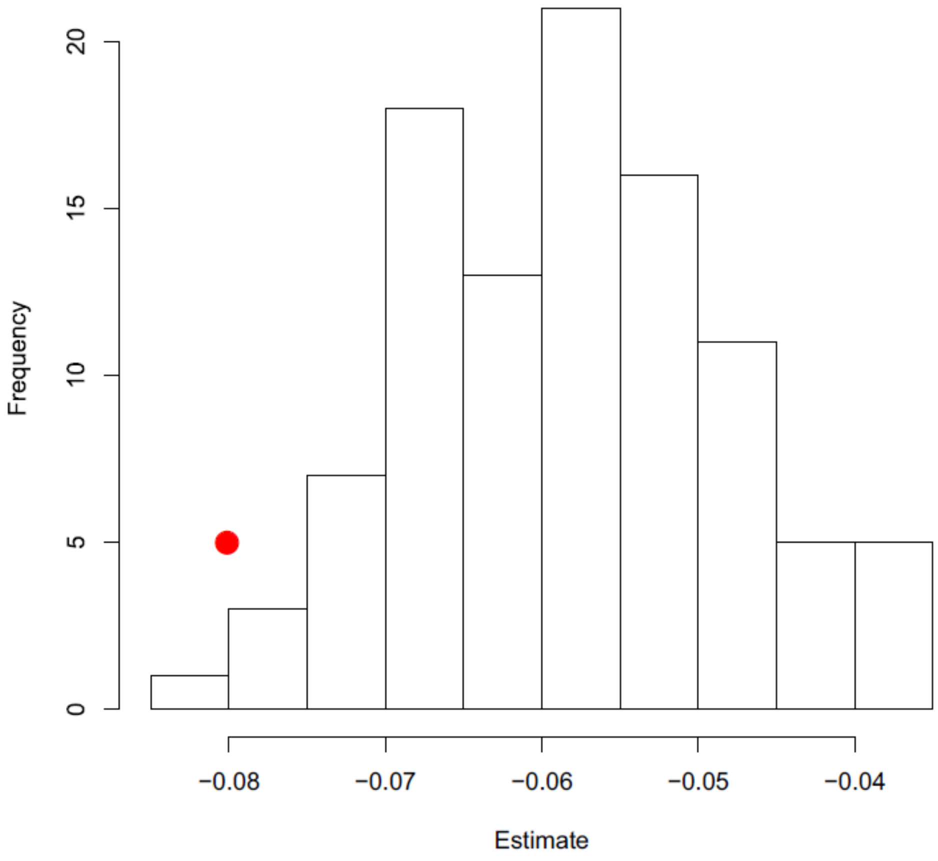

Figure S2. Distribution of score effect sizes from random exon permutations.

A histogram of the score effect size estimates from 100 random exon permutations. The true exon division score effect size estimate is marked as a red circle.

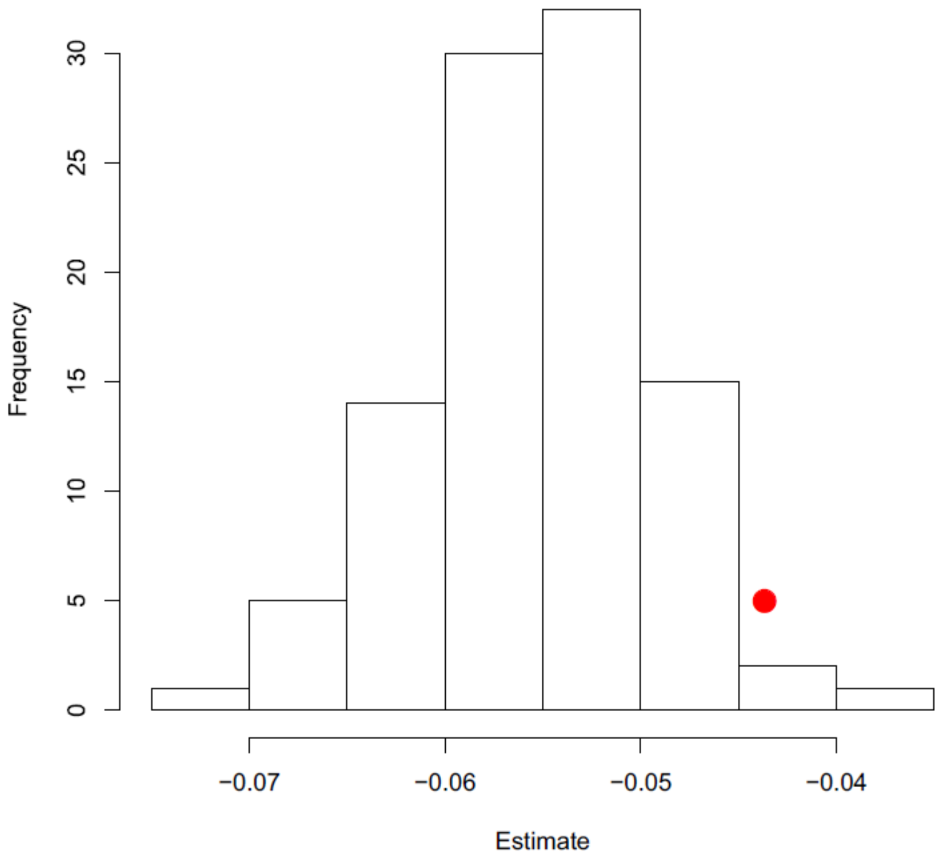

Supplement: Additional file 6: — A PDF containing Figure S1 and Figure S2, which show the distribution of score effect sizes from random permutations. (PDF 195 kb) [file 13059_2016_869_MOESM6_ESM.pdf]
