# Supplementary material for: The intolerance to functional genetic variation of protein domains predicts the localization of pathogenic mutations within genes
Source: Genome Biol. 2016 Jan 18;17:9. doi: 10.1186/s13059-016-0869-4 (PMC4717634; doi:10.1186/s13059-016-0869-4)
Supplement: Additional file 8: — A PDF containing the full results of the comparison to variant level predictors. (PDF 88 kb) [file 13059_2016_869_MOESM8_ESM.pdf]

Examining the Relationship with Variant Level Scores

We generated a set of 250,000 simulated variants (Additional file 7) within the domain subRVIS coordinates. Each domain sub region had an equal likelihood of a simulated variant falling within its boundaries. This was in order to ensure balanced representation across the domain sub regions. For each variant level scoring methodology (PolyPhen-2 [1], CADD [2], MutationTaster [3]) we calculated the corresponding *in silico* variant prediction. In each methodology, if a variant had multiple predictions, it was assigned the most damaging prediction. We then assigned each of these variants a subRVIS score corresponding to the subRVIS score of the domain sub region it falls in. If a variant fell within the boundaries of overlapping genes and therefore more than one domain sub region, it was assigned the most intolerant domain subRVIS score across all the domain sub regions it falls in. Following this, we examined the relationship between each of the methodologies' scores and the subRVIS scores.

PolyPhen-2

PolyPhen-2 [1] is a variant level predictor, which predicts the effect of a variant on the function of a protein. We calculated the PolyPhen-2 HumVar probabilities across all the simulated variants, where available. Higher PolyPhen-2 probabilities indicate more damaging predictions. We calculated the correlation between the PolyPhen-2 scores and the subRVIS scores. We found that the Pearson's correlation coefficient between these two sets of scores is -0.0548 (p-value: $<2.2\times10^{-16}$; 95% confidence interval: [-0.0595, -0.0501], 173,879 variants). The negative correlation is expected, as lower subRVIS scores indicate more intolerant regions and higher PolyPhen-2 scores indicate more damaging variants. However, the low correlation suggests that the scores do provide independent information.

CADD

CADD [2] is a variant level predictor, which predicts the deleteriousness of a variant based on the integration of multiple metrics. We calculated the raw CADD scores across all the simulated variants, where available. Higher CADD scores are predicted to be more deleterious. We calculated the correlation between the CADD scores and the subRVIS scores. We found that the Pearson's correlation coefficient between these two sets of scores is -0.0811 (p-value: $<2.2\times10^{-16}$; 95% confidence interval: [-0.0850, -0.0772], 249,446 variants). As with PolyPhen-2, the negative correlation is expected, as lower subRVIS scores indicate more intolerant regions and higher CADD scores indicate more deleterious variants. However, as with PolyPhen-2, the correlation is low.

MutationTaster

MutationTaster [3] is a variant level Bayes classifier that predicts whether a variant is disease causing or not. MutationTaster takes multiple metrics into account, including protein domain information when it is available. We calculated the MutationTaster scores across all the simulated variants, where available. We converted the predictions into scores on a scale of zero to one, with zero corresponding to predicted pathogenic and one corresponding to predicted non-pathogenic (Methods). Following this, we calculated the correlation between the MutationTaster scores and the subRVIS scores.

We found that the Pearson's correlation coefficient between these two sets of scores is 0.159 (p-value: $<2.2\times10^{-16}$; 95% confidence interval: [0.155, 0.163], 248,079 variants). Thus, there does appear to be positive correlation between domain subRVIS and MutationTaster, to a higher degree than between subRVIS and either of the other two scores (PolyPhen-2 and CADD).

References

1. Adzhubei IA, Schmidt S, Peshkin L, Ramensky VE, Gerasimova A, Bork P et al. A method and server for predicting damaging missense mutations. Nat Methods. 2010;7(4):248-9. doi:10.1038/nmeth0410-248.

2. Kircher M, Witten DM, Jain P, O'Roak BJ, Cooper GM, Shendure J. A general framework for estimating the relative pathogenicity of human genetic variants. Nat Genet. 2014;46(3):310-5. doi:10.1038/ng.2892.

3. Schwarz JM, Cooper DN, Schuelke M, Seelow D. MutationTaster2: mutation prediction for the deep-sequencing age. Nat Methods. 2014;11(4):361-2. doi:10.1038/nmeth.2890.
